# Recombinant Protein Containing B-Cell Epitopes of Different *Loxosceles* Spider Toxins Generates Neutralizing Antibodies in Immunized Rabbits

**DOI:** 10.3389/fimmu.2018.00653

**Published:** 2018-04-03

**Authors:** Sabrina de Almeida Lima, Clara Guerra-Duarte, Fernanda Costal-Oliveira, Thais Melo Mendes, Luís F. M. Figueiredo, Daysiane Oliveira, Ricardo A. Machado de Avila, Valéria Pereira Ferrer, Dilza Trevisan-Silva, Silvio S. Veiga, João C. Minozzo, Evanguedes Kalapothakis, Carlos Chávez-Olórtegui

**Affiliations:** ^1^Departamentos de Bioquímica-Imunologia, Universidade Federal de Minas Gerais, Belo Horizonte, Brazil; ^2^Programa de Pós-Graduação em Ciências da Saúde – PPGCS, Universidade do Extremo Sul Catarinense – UNESC, Criciúma, Brazil; ^3^Universidade Federal de Paraná, Curitiba, Brazil; ^4^Centro de Produção e Pesquisa de Imunobiológicos – CPPI, Piraquara, Brazil; ^5^Departamentos de Biologia Geral, Universidade Federal de Minas Gerais, Belo Horizonte, Brazil

**Keywords:** *Loxosceles* spider venom, sphingomyelinase D, astacin-like metalloprotease hyaluronidase, multiepitopic recombinant protein, B-cell epitope

## Abstract

Loxoscelism is the most important form of araneism in South America. The treatment of these accidents uses heterologous antivenoms obtained from immunization of production animals with crude loxoscelic venom. Due to the scarcity of this immunogen, new alternatives for its substitution in antivenom production are of medical interest. In the present work, three linear epitopes for Loxosceles astacin-like protease 1 (LALP-1) (SLGRGCTDFGTILHE, ENNTRTIGPFDYDSIMLYGAY, and KLYKCPPVNPYPGGIRPYVNV) and two for hyaluronidase (LiHYAL) (NGGIPQLGDLKAHLEKSAVDI and ILDKSATGLRIIDWEAWR) from *Loxosceles intermedia* spider venom were identified by SPOT-synthesis technique. One formerly characterized linear epitope (DFSGPYLPSLPTLDA) of sphingomyelinase D (SMase D) SMase-I from *Loxosceles laeta* was also chosen to constitute a new recombinant multiepitopic protein. These epitopes were combined with a previously produced chimeric multiepitopic protein (rCpLi) composed by linear and conformational B-cell epitopes from SMase D from *L. intermedia* venom, generating a new recombinant multiepitopic protein derived from loxoscelic toxins (rMEPLox). We demonstrated that rMEPLox is non-toxic and antibodies elicited in rabbits against this antigen present reactivity in ELISA and immunoblot assays with Brazilian *L. intermedia, L. laeta, L. gaucho*, and *L. similis* spider venoms. *In vivo* and *in vitro* neutralization assays showed that anti-rMEPLox antibodies can efficiently neutralize the sphingomyelinase, hyaluronidase, and metalloproteinase activity of *L. intermedia* venom. This study suggests that this multiepitopic protein can be a suitable candidate for experimental vaccination approaches or for antivenom production against *Loxosceles* spp. venoms.

## Introduction

Loxoscelism is the most important form of araneism in South America. It constitutes the first cause of accidents by venomous animals in Peru and Chile (1, 2). In Brazil, more than 7,000 human cases of loxoscelism occur annually [(3)—SINAN]. Clinical manifestations of loxoscelism include local cutaneous and systemic forms. Local effects are commonly observed near the bite site, which is characterized by skin necrosis and slow progression ulceration. Systemic envenoming occurs in approximately 10% of the cases, depending on the species involved. Systemic symptoms include acute renal failure, intravascular hemolysis, thrombocytopenia, and disseminated intravascular coagulation (4).

Sphingomyelinase D (SMase D, also called phospholipase D), Loxosceles astacin-like proteases (LALPs), and hyaluronidases (HYALs) are some of the main components expressed in *Loxosceles* spp. venom glands responsible for human envenoming symptoms (5, 6). SMases D can cause platelet aggregation, intravascular hemolysis and are mainly responsible for dermonecrosis development, the most typical sign of loxoscelism (7–9). HYAL seems to be responsible for increasing venom diffusion and gravitational spreading of the lesion (10–12). LALPs were characterized for their proteolytic action, suggesting that they act in venom spreading and in local hemorrhage (13).

Monoclonal or polyclonal antibodies have been produced against crude loxoscelic venoms and are able to recognize SMases D, HYALs, and metalloproteases, which demonstrates the antigenic potential of these proteins (12, 14–17). In addition, synthetic peptides corresponding to epitopes from SMase D from *Loxosceles intermedia* venom (LiD1) induced antibody responses that efficiently neutralized the venom toxic effects (18–21).

These previous results suggest that synthetic and non-toxic immunogens could be used to produce therapeutic antivenoms or in vaccinations for loxoscelic envenoming. Combining this prior knowledge, in 2013, our group synthetized a chimeric recombinant protein (rCpLi) containing three epitopes previously mapped in SMase LiD1, from *L. intermedia* (22). Immunization with rCpLi produced antibodies with potential of neutralizing toxic effects of *L. intermedia* venom. We believe that this strategy can be improved by the generation of a immunogen containing a recombinant or synthetic multi-epitope proteins, consisting of linear and conformational epitopes not only from SMase D, but also from other major toxins of *Loxosceles* spp. venoms that act in envenoming.

There are different approaches that could be employed to map B cell epitopes, such as computational-based methods, surface plasmon resonance, high-density peptide microarray epitope mapping, and SPOT synthesis (23–25). The SPOT synthesis is an economically viable technique that allows the evaluation of several peptides simultaneously and for that reason it was more suitable for our purpose. In this work, we report the localization of linear B-cell epitopes, using the SPOT method of multiple peptide synthesis (25, 26) on astacin-like protease (LALP-1) and hyaluronidase (LiHYAL) toxins from *L. intermedia* venom.

After epitope identification, we show the design and production of a multiepitopic recombinant, non-toxic protein (rMEPLox) containing epitopes from LALP-1, LiHYAL as well as from SMase D from *L. intermedia* and from SMase-I from *Loxosceles laeta* venoms. Immunization studies in rabbits with rMEPLox as immunogen suggests that this multiepitopic protein can be a suitable candidate to produce antivenoms or vaccines against *Loxosceles* spp. spider venoms.

## Materials and Methods

### Animals, Venoms, and Sera

BALB/c mice (18–22 g) and New Zealand rabbits (2 kg) were obtained and maintained at Centro de Bioterismo of Instituto de Ciências Biológicas and of Escola de Medicina Veterinária of Universidade Federal de Minas Gerais (UFMG), respectively. All animals received food and water *ad libitum*, under controlled environmental conditions. Experimental protocols were performed after approval by the Ethics Committee in Animal Experimentation of UFMG (388/2017-CETEA/UFMG).

*Loxosceles intermedia, L. laeta*, and *L. gaucho*, venoms samples were provided by Dr. João Carlos Minozzo from Centro de Pesquisa e Produção de Imunobiologicos of Paraná State (CPPI). *L. similis* and *L. laeta* (Peru) venoms were provided by Dr. Evanguedes Kalapothakis from UFMG and by Dr. Cesar Bonilla from Instituto Nacional de Salud of Peru, respectively. The lyophilized samples were stored at −20°C in the dark until use.

Rabbit sera developed against the recombinant form of LALP-1 and LiHYAL (used to map epitopes within their sequences) were provided by the team of Dr. Silvio Sanchez Veiga from Universidade Federal do Paraná.

### Identification of Epitopes in LALP-1 and LiHYAL

#### Peptide Synthesis on Cellulose Membranes

Eighty-four overlapping pentadecapeptides (15-mer), frameshifted by three residues, covering the amino acid sequences of LALP-1 (UniProt: A0FKN6.1) (13) and 130 15-mer overlapping peptides from LiHYAL (UniProt: R4J7Z9) (12), were prepared using the SPOT-technique (25), following the protocol of Laune et al. (27). Cellulose membranes were obtained from Intavis (Koln, Germany); fluorenylmethyloxycarbonyl amino acids and *N*-hydroxybenzotriazole were from Novabiochem. A Multipep ResPep SL Automatic SPOT-synthesizer (IntavisAG, Bioanalytical Instruments, Germany) was used for automated peptide synthesis in the membrane. After peptide sequences were assembled, the side-chain protecting groups were removed by treatment with trifluoroacetic acid.

#### Immunoassay With Cellulose-Bound Peptides

After an overnight saturation step with 3% BSA, the membrane containing peptides from LALP-1 or from LiHYAL was probed by incubation with rabbit serum anti-LALP-1r and anti-LiHYALr (diluted 1/200), respectively. Antibody binding was detected using alkaline–phosphatase conjugated anti-horse or anti-rabbit antibody (Sigma, diluted 1/3,000; 90 min, 37°C). After washing, phosphatase substrate [5-bromo-4-chloro-3-indolyl phosphate and 3-(4,5-dimethylthiazol-2-yl)-2,5 diphenyltetrazolium bromide, both from Sigma] was added. A blue precipitate was observed over the spots that reacted with the tested antibodies. The membrane was placed on a color scanner and scanned without scale reduction. All spots were analyzed using ImageJ software, and a cutoff of 600,000 pixels was defined. To allow membrane reuse, it was sequentially treated with dimethylformamide, then 1% SDS, 0.1% 2-mercaptoethanol in 8 M urea, then ethanol/water/acetic acid (50:40:10 vol/vol/vol) and, finally, ethanol, to remove the precipitated dye and molecules bound to peptides.

### Design and Construction of Recombinant Multiepitopic Protein (rMEPLox)

rMEPLox was designed and constructed by adding newly mapped epitopes to a chimeric protein (rCpLi) formerly characterized by our group (22). rCpLi contains three epitopes of SMase D LiD1, from *L. intermedia*, mapped in previous studies (NLGANSIETDVSFDDNANPEYTYHGIP, SKKYENFNDFLKGLR and NCNKNDHLFACW) (19, 21). One epitope (DFSGPYLPSLPTLDA) of SMase-I from *L. laeta*, characterized by Ramada et al. (28), together with three epitopes of LALP-1 (SLGRGCTDFGTILHE, ENNTRTIGPFDYDSIMLYGAY and KLYKCPPVNPYPGGIRPYVNV) and two epitopes of LiHYAL (NGGIPQLGDLKAHLEKSAVDI and ILDKSATGLRIIDWEAWR), mapped in the present work, were added to rCpLi sequence, forming the new chimeric multiepitopic protein rMEPLox.

Amino acid sequence of the defined epitopes was converted to DNA nucleotide sequence, using two glycine codons as spacers between each epitope (22). The nucleotide sequence of the new selected epitopes was optimized and synthesized by GenScript company and inserted into the pET expression vector, already containing the sequence of rCpLi (22). Sequences were confirmed by Sanger sequencing, performed on the ABI3130 Applied Biosystems sequencer, using capillary electrophoresis with POP7 polymer and the BigDye v3.1 fluorescent terminator. *E. coli* BL21 bacteria were used for transformation and its efficiency was confirmed by colony PCR.

### Expression and Purification of rMEPLox

A positive colony was selected for expression in 2xYT culture medium supplemented with 50 μg/mL kanamycin. The culture was incubated at 37°C, stirring at 180 rpm and cell growth was controlled to an OD600 of 0.4. Expression was then induced by the addition of IPTG, at a final concentration of 0.6 mM and continued for 4 h. Cells were resuspended in lysis buffer (50 mM Tris, 500 mM NaCl, 20 mM imidazole, 5 mM MgSO_4_, pH 7.4), and lysozyme was added to a final concentration of 100 μg/mL. The extract was subjected to three cycles of freezing in liquid nitrogen and thawing at 37°C. After that, bacteria were sonicated for total release of cytoplasmic proteins. The suspension was then centrifuged at 9,000 *g* at 4°C for 30 min. Bacterial supernatant and pellet were submitted to polyacrylamide gels electrophoresis. A higher concentration of rMEPLox was found in the insoluble extract. Thus, the extract pellet was resuspended in binding buffer (8 M urea, 50 mM Tris, 500 mM NaCl, 20 mM imidazole, 5 mM MgSO_4_, pH 7.4) added with 10 mM PMSF solution. Purification of recombinant rMEPLox was done by affinity chromatography using HP HisTrap™ 5 mL column on ÄKTAprime plus (GE Healthcare) according to manufacturer’s specifications.

### Enzymatic Activities

Sphingomyelinase activity was determined using the Amplex^®^ Red sphingomyelinase assay kit (Molecular Probes, Invitrogen, USA), according to manufacturer’s instructions. The concentrations of 1 μg of *L. intermedia* venom and 30, 15, and 7.5 μg of rMEPLox were tested. Hydrolysis of sphingomyelin was detected indirectly by measuring fluorescence with the fluorescence micro plate reader (Varian, Agilent Technologies, CA, USA), using excitation at 530 nm and detection at 590 nm.

Hyaluronidase activity was determined using the method described by Horta et al. (29) with modifications: concentrations ranging from 0.3 to 40 μg of *L. intermedia* crude venom or rMEPLox were incubated with 0.2 M acetate buffer, 15 M NaCl, pH 6, and 2.5 mg hyaluronic acid (HA)/mL in 96-well Falcon microplates (Becton Dickinson, France). A curve with different concentrations of HA was used as control, corresponding to values of 100, 75, 50, 25, and 0% of HYAL activity. The plate was incubated at 37°C for 30 min. Subsequently, 200 μL of 2.5% (w/v) cetyltrimethylammoniumbromide (CTAB) dissolved in 2% (w/v) NaOH was added to the wells for HA precipitation. The produced turbidity was measured at 405 nm. The assays were performed in duplicates and concentrations corresponding to 50% of HYAL activity for crude venom or rMEPLox were determined.

Metalloproteinase activity of *L. intermedia* venom and rMEPLox was determined by fibrinogen (Fg) digestion, as described by Bello et al. (30) Fg was dissolved at a final concentration of 2.5 mg/mL in 25 mM Tris–HCl buffer (pH 7.4) containing 0.15 M NaCl. *L. intermedia* venom (3 μg) or rMEPLox (3 μg) were added to 0.5 mL of Fg solution at a molar ratio of enzyme to substrate of 1/52 (w/w). The mixture was incubated at 37°C for 16 h. After this period, 50-μL aliquots were mixed with an equal volume of denaturing solution (10 M urea, 4% β-mercaptoethanol, 4% SDS) for a further 16 h. A 15-μL aliquot of the final solution was applied in 10% SDS-PAGE under reducing conditions and stained with Coomassie.

### Antigenic Analysis of rMEPLox by ELISA

Antigenic cross-reactivity of rMEPLox with antivenom sera produced against different loxoscelic venoms [*L. intermedia, L. gaucho, L. laeta* (Brazil), *L. laeta* (Peru)] or anti-individual toxins (rLALP-1, rLiHYAL, rLiD1) antibodies was determined by ELISA as described by Chavez-Olortegui et al. (31). Absorbance values were determined at 490 nm with Biorad 680 plate reader. All measurements were made in duplicates.

### Immunization Protocols

Adult female rabbits were used for vaccination approaches and production of anti-rMEPLox antibodies. After collection of pre-immune (PI) sera, a group of two animals received an initial subcutaneous (s.c.) injection of 200 μg of rMEPLox in Montanide adjuvant (day 1). Three booster injections (s.c.) were administered at intervals of 2 weeks with the same rMEPLox dose (200 μg) in Montanide adjuvant. For production of anti-loxoscelic venom polyvalent antibodies, two additional rabbits received an initial dose of 21 μg (s.c.) of a mixture containing *L. gaucho, L. intermedia*, and *L. laeta* venoms (7 μg of each) in Montanide adjuvant. Three subcutaneous booster injections of the same dose in Montanide were made, at intervals of 2 weeks. Blood samples were collected 1 week after the third dose. After a break of 3 months, rabbits received three additional doses of either 200 μg of rMEPLox or 21 μg of loxoscelic venom mixture in Montanide adjuvant at intervals of 2 weeks. Blood samples were obtained 1 week and 3 months after last injection. As negative control, two rabbits received only Montanide adjuvant, following the same protocol for the immunized groups. IgGs were purified from the anti-loxoscelic or anti-rMEPLox sera by precipitation with ammonium sulfate followed by affinity chromatography with a Protein A-Sepharose column (GE Healthcare), according to the protocol described by GE Healthcare Bio-Sciences AB.

### Immunoassays to Detect Anti-rMEPLox Antibodies

#### Indirect ELISA

MaxiSorp plates purchased from NUNC were coated overnight at 4°C with 100 μL of a 5 μg/mL solution of either *L. intermedia, L. gaucho, L. similis*, or *L. laeta* venoms or rMEPLox in 0.02 M sodium bicarbonate buffer, pH 9.6. After blocking [1% skimmed non-fat milk in phosphate buffer saline (PBS)] and washing (0.05% Tween 20-PBS), sera from PI and immune rabbits were added in serial dilution from 1/400 to 1/256,000 and incubated for 1 h at 37°C. Plates were washed and incubated with anti-rabbit IgG conjugated with horseradish peroxidase (HRP, Sigma-Aldrich A9292) diluted 1/4,000, for 1 h at 37°C. ELISA was carried out as described by Chavez-Olortegui et al. (31). Absorbance values were determined at 490 nm using an ELISA plate reader (BIO-RAD, 680 models). Duplicate assays were taken for all samples and means calculated.

#### Electrophoresis and Western Blotting

Samples containing 10 μg of each *Loxosceles* venom were solubilized in reducing sample buffer and subjected to SDS-PAGE on 15% polyacrylamide gels. After electrophoresis, gels were transferred onto nitrocellulose membranes. Membranes were blocked with blocking buffer (1% skimmed non-fat milk 0.3% Tween 20-PBS) for 1 h and then incubated with sera from immunized rabbits, diluted in the same blocking buffer (1/200) for 1 h at room temperature (RT). Membranes were washed with 0.05% Tween 20-PBS and incubated with goat anti-rabbit IgG labeled with HRP (diluted 1/4,000) in blocking buffer for 1 h at RT. Membranes were washed once again and blots were developed using 3,3′-diaminebenzidine tetrahydrochloride hydrate (DAB) plus 4-chloro-1-naphthol as previously described (17).

### *In Vivo* Neutralization Assays

Dermonecrosis caused by *L. intermedia* crude venom was determined in immunized rabbits, according to Furlanetto et al. (32). Ninety days after the last immunization dose, rabbits from each group (rMEPLox, Venom or C-) received an intradermal injection of 10 μg of *L. intermedia* venom (corresponding to 3.35 MND—Minimum Necrotizing Dose, which was previously determined in CPPI with the same lot of venom), on the shaved back skin. Animals were observed for 72 h. After this period, measurements of the affected area at the injection site area were taken.

For neutralization of lethal activity of *L. intermedia* venom in mice, three groups of five animals were used. The median lethal dose (LD_50_) for *L. intermedia* venom was previously determined as 10 μg/20 g of mice by Braz et al. (33). Each mouse received intraperitoneally (i.p.) 0.5 mL of a solution containing 2.5 LD_50_ of *L. intermedia* venom in PBS, pre-incubated for 1 h at 37°C with either with 100 μL of anti-rMEPLox sera (Group 1), 100 μL of PI rabbit sera (Group 2), or 100 μL of PBS (Group 3). Animals were observed 24, 48, and 72 h after the injection. After this period, deaths were counted.

### *In Vitro* Neutralization Assays

The ability of anti-rMEPLox antibodies to inhibit the bovine Fg digestion by *L. intermedia* venom (metalloproteinase activity) was tested as described by Bello et al. (30). Briefly, IgG anti-rMEPLox (2, 10, 40, and 100 μg) was incubated with *L. intermedia* venom (3 μg) in PBS for 1 h at 37°C. Then, the assay proceeded as previously described in Section “Enzymatic Activities.” Experimental control was performed with Fg alone in the same conditions. Negative controls were performed with IgGs from non-immune rabbits for all the three assays.

To neutralize the *in vitro* HYAL activity of *L. intermedia* venom, 2 μL of anti-rMEPLox serum were incubated with *L. intermedia* venom (1 LD_50_ = 6.6 μg) for 1 h at 37°C before the turbidimetric assays were performed. Then, the turbidimetric assay was performed as previously described, with some volumetric modifications. The assay mixture contained 12.5 μg of HA (Sigma-Aldrich), acetate buffer (0.2 M sodium acetate–acetic acid pH 6.0, 0.15 M NaCl), and test sample in a final volume of 250 μL. Subsequently, 500 μL of 2.5% (w/v) CTAB dissolved in 2% (w/v) NaOH was added to the wells for HA precipitation. Negative control was made with non-immune serum (2 μL) and the commercial serum of CPPI (2 μL) was used as positive control. Three independent experiments were performed in triplicate.

## Results

### Identification of Epitopes in LALP-1 and LiHYAL

**Figure 1:**
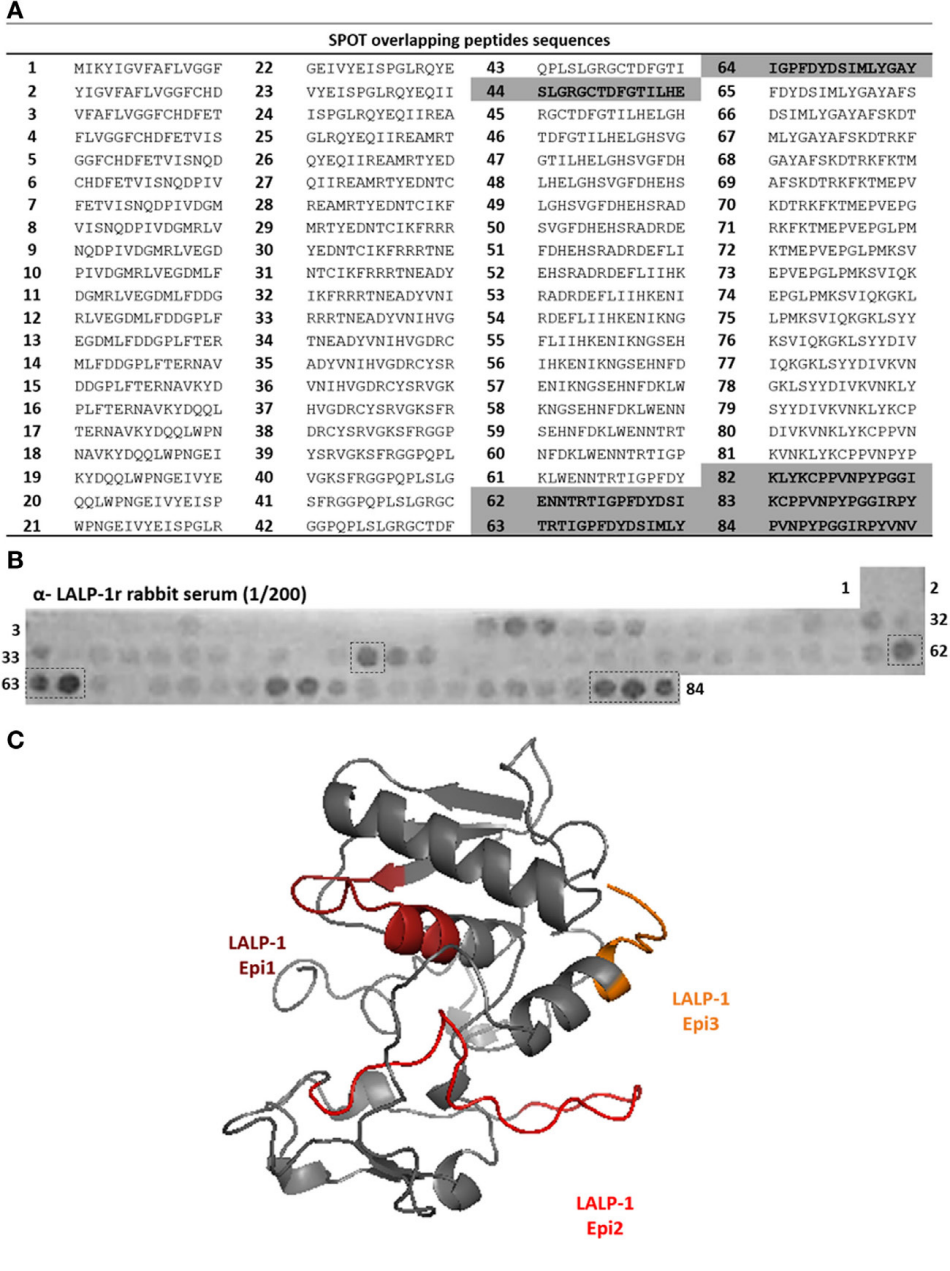
Epitope mapping of LALP-1 by SPOT method. **(A)** List of peptides sequences derived from LALP-1 sequence synthetized bound to the membrane, according to spot number. Sequences of most reactive spots are highlighted in gray. **(B)** Cellulose membrane containing bound 15-mer overlapping peptides derived from LALP-1 sequence, probed with anti-rLALP-1 rabbit serum, diluted 1/200. After incubation with secondary anti-rabbit-alkaline phosphatase antibody, MTT-BCIP substrate was added, revealing reactive spots in the membrane. Spots of higher intensity measured by ImageJ software are marked by a dotted line. **(C)** Homology model of LALP-1, generated by the I-TASSER server (34), using bone morphogenetic protein 1 protease domain, from *Homo sapiens* (PDB: 3EDH) as template. The epitope sequences mapped in the membrane are marked in orange, red andburgundy in the modeled structure.

To map linear B-cell epitopes on LALP-1, we prepared 84 overlapping 15-mer peptides (Figure 1A) covering the complete amino acid sequence of the enzyme by the SPOT method (25). Figure 1B shows the binding pattern of anti-LALP-1r rabbit sera (1/200 dilution) to the membrane-bound peptides. Several peptides were recognized by antibodies: in the central region (peptide 44) and in the C-terminal part of the protein (peptides 62–64 and 82–84). Weaker binding was observed on other peptides (20–24 and 71–72), consistent with the polyclonality of the used antibodies. Binding between anti-LALP-1r sera antibodies and membrane-bound peptides seems to be specific since neither PI serum or alkaline phosphatase labeled anti-rabbit antibody showed reactivity with the membrane (data not shown). Residues 114–128, residues 165–188, and residues 227–248, corresponding, respectively, to peptides 44, 62–64, and 82–84, were found to be surface exposed on the three-dimensional model of this enzyme (Figure 1C), consistent with their antigenic recognition by anti-LALP-1r polyclonal antibodies. Thus, the amino acid sequence of peptides corresponding to continuous epitopes in the central part (SLGRGCTDFGTILHE, residues 114–128) and C-terminal (ENNTRTIGPFDYDSIMLYGAY, residues 165–188 and KLYKCPPVNPYPGGIRPYVNV residues 227–248) regions were chosen to integrate the multiepitopic protein rMEPLox.

**Figure 2:**
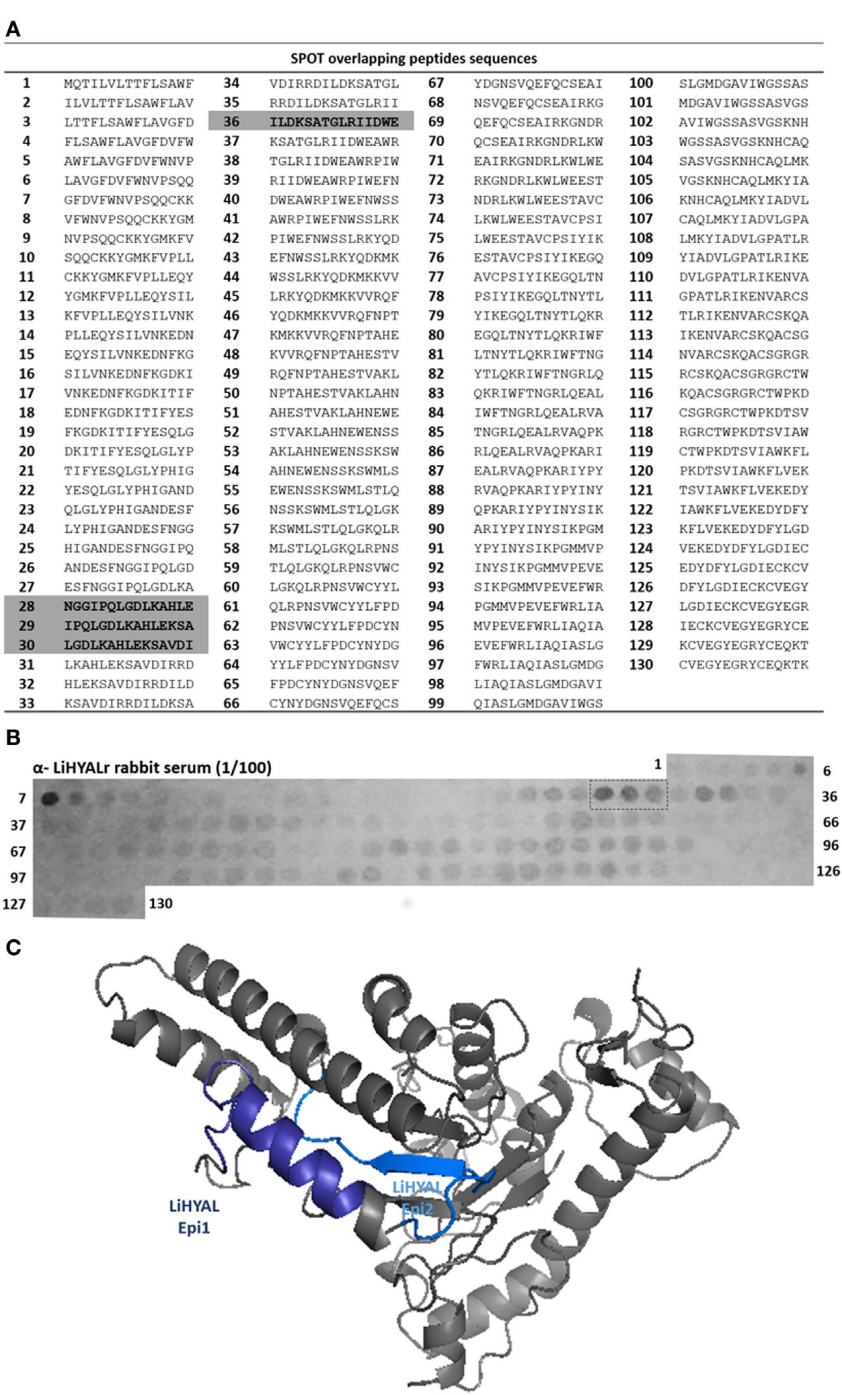
Epitope mapping of LiHYAL by SPOT method. **(A)** List of peptides sequences derived from LiHYAL sequence synthetized bound to the membrane, according to spot number. Sequences of most reactive spots and the chosen peptides containing the active site are highlighted in gray. **(B)** Cellulose membrane containing bound 15-mer overlapping peptides derived from LiHYAL sequence, probed with anti-rLiHYAL rabbit serum, diluted 1/100. After incubation with secondary anti-rabbit-alkaline phosphatase antibody, MTT-BCIP substrate was added, revealing reactive spots. Spots of higher intensity (measured by ImageJ software) are marked by a dotted line. **(C)** Homology model of LiHYAL, generated by the I-TASSER server (34), using hyaluronidase (HYAL) 1, from *Homo sapiens* (PDB: 2PE4) as template. The epitope sequence mapped in the membrane are marked in dark blue and active site in light blue, in the modeled structure.

To map epitopes on LiHYAL, we prepared 130 overlapping 15-mer peptides covering the amino acid sequence of LiHYAL (Figure 2A). Figure 2B shows the pattern of binding of anti-LiHYALr rabbit sera to membrane-bound peptides. Differently from LALP-1, few peptides (two) were recognized by these antibodies in the N-term region (peptides 6–7 and peptides 28–30). Peptides 6 and 7 had amino acids pertaining to the signal peptide of LiHYAL in its sequence and were, therefore, excluded from our analysis. Peptides 28–30 (NGGIPQLGDLKAHLEKSAVDI, residues 63–83) and peptides 36 and 37 (DIRRDILDKSATGLRIIDWE, residues 87–101) which correspond to the active part of the enzyme were also localized on the HYAL three-dimensional model (Figure 2C) and were chosen to compose the multiepitopic protein rMEPLox.

### Design, Expression, and Purification of rMEPLox

**Figure 3:**
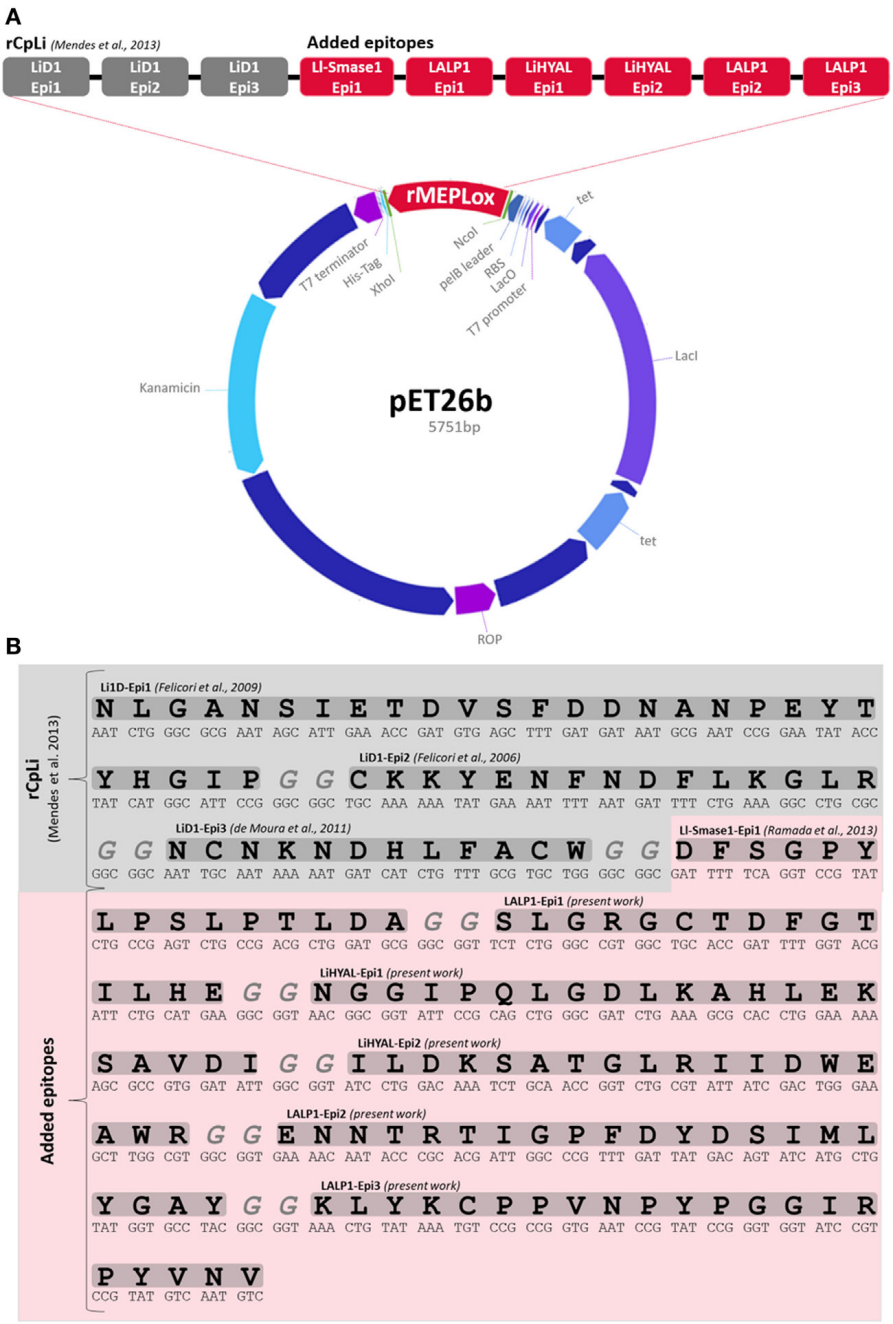
Structure of rMEPLox. **(A)** pET 26 vector structure, showing its main components and the location of rMEPLox and epitope composition of rMEPLox. The first three epitopes from LiD1 in gray composed the previously produced chimeric protein rCpLi. The newly mapped epitopes are marked in red and were placed in the demonstrated order. **(B)** rMEPLox amino acid and nucleotidic sequence.

rMEPLox was constructed containing three epitopes from SMase D of *L. intermedia* (NLGANSIETDVSFDDNANPEYTYHGIP, SKKYENFNDFLKGLR, and NCNKNDHLFACW), one epitope (DFSGPYLPSLPTLDA) of *L. laeta* SMase-I, three epitopes from LALP-1 (SLGRGCTDFGTILHE, ENNTRTIGPFDYDSIMLYGAY, and LYKCPPVNPYPGGIRPYVNV), one epitope from LiHYAL (NGGIPQLGDLKAHLEKSAVDI) and finally the peptidic sequence (ILDKSATGLRIIDWEAWR) corresponding to the active center of LiHYAL, disposed as shown in Figure 3A. A spacer with two glycine codons between the epitope sequences was added. The sequences were cloned into pET 26b (+) vector, containing His6-tag and signal sequence for expression in the periplasm (pelB leader). The map of the expression vector produced by the Gene Design program can be visualized below in Figure 3A. The construction totaled 5,751 bp and was synthesized by GenScript. The amino acid and nucleotidic sequence of rMEPLox is shown in Figure 3B.

**Figure 4:**
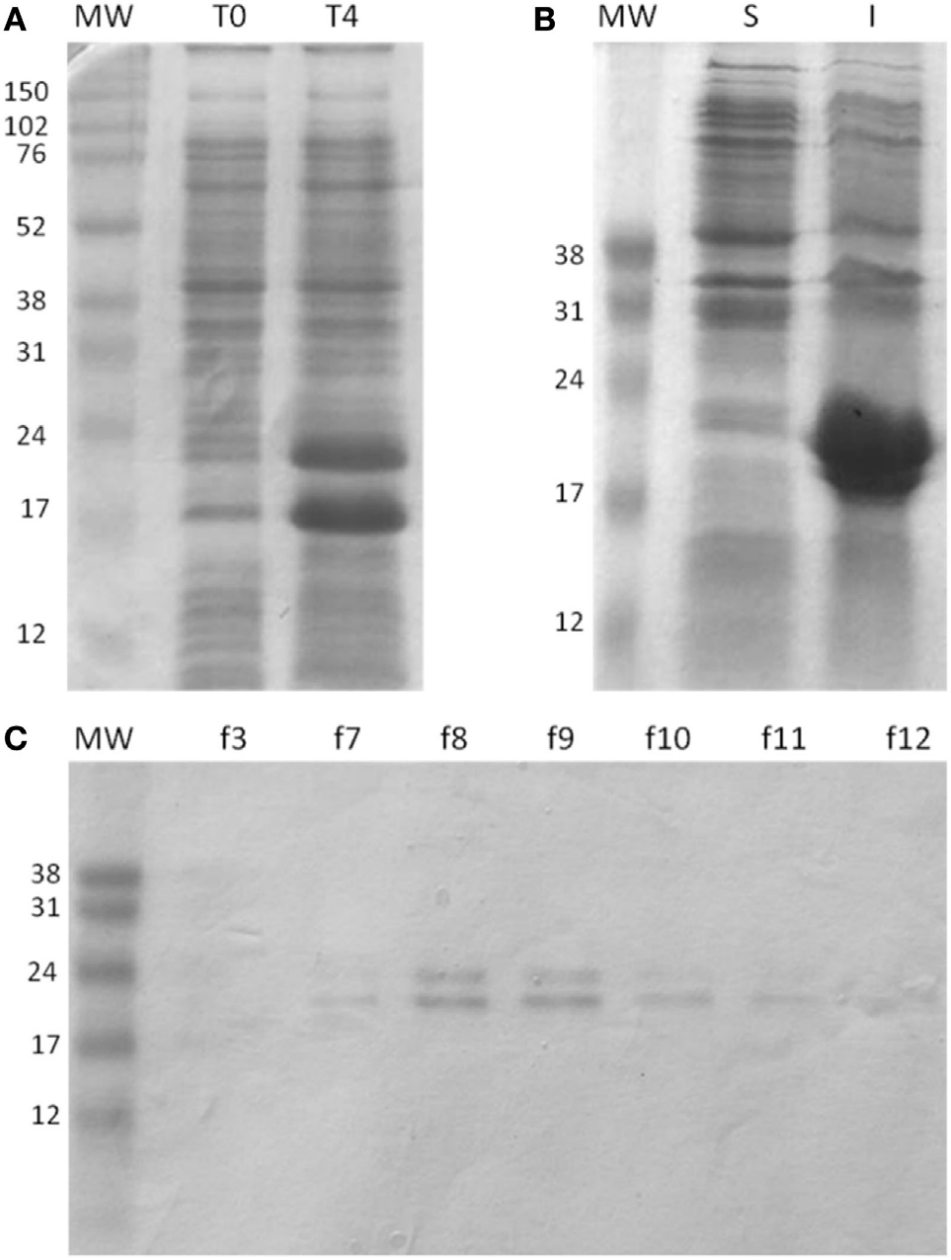
Expression and purification of rMEPLox. The protein was analyzed by SDS/PAGE (15% gel) under reducing conditions and Coomassie Blue staining. **(A)** Expression of rMEPLox. T0: negative control without induction by IPTG; T4: the expression of rMEPLox induced by IPTG (0.6 mM at 37°C for 4 h). **(B)** Solubility analysis of the induced expression of rMEPLox. S: clarified supernatant of the induced recombinant pET-26b-rMEPLox culture; I: insoluble pellet of the induced recombinant pET-26b-rMEPLox culture. **(C)** Purification in HisTrapTM HP of rMEPLox. Each lane corresponds to a fraction obtained in the purification. MW: protein marker.

The expression of the chimeric gene in pET 26b vector was performed in *E. coli* strain BL21 and produced after 4 h of induction with IPTG. The constructed rMEPLox has a predicted mass of 19.39 kDa and isoelectric point of 5.10, as defined by Prot-Param tool of ExPASy portal (35). After expression, it was possible to visualize in the SDS-PAGE the increase in expression of two protein bands, between 24 and 17 kDa, which would be compatible with rMEPLox with and without the pelB leader signal sequence, respectively (Figure 4A). After cytoplasmic lysis, the recombinant protein was present at a higher concentration in the pellet (Figure 4B), indicating that the protein was expressed largely in an insoluble form. The bacterial extract obtained was purified using HP HisTrap ™ column (GE Healthcare). Through SDS-PAGE of the fractions obtained from purification, it can be seen the elimination of most unrelated proteins, obtaining only bands from rMEPLox expression (Figure 4C).

### Antigenic and Toxic Characterization of rMEPLox

**Figure 5:**
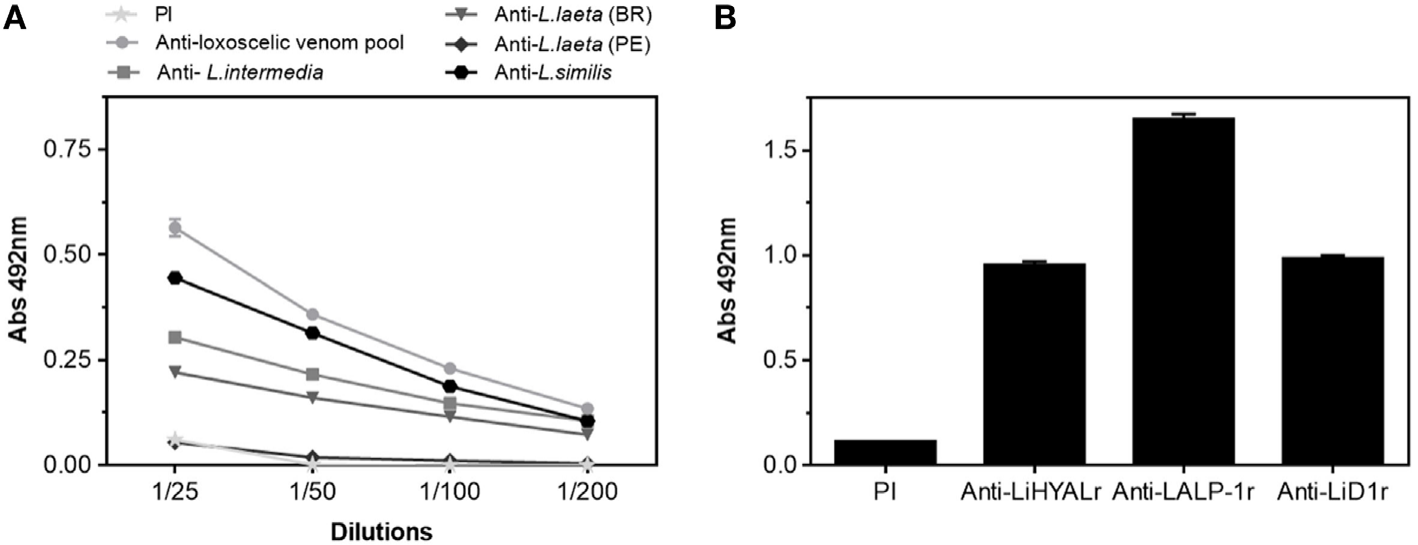
Immunoreactivity of the rMEPLox by ELISA. The plates were coated with rMEPLox 5 μg/mL. Pre-immune (PI) sera was used as negative control. **(A)** ELISA showing the reaction of rMEPLox with antibodies developed against a pool of loxoscelic venoms or individual venoms from *L. intermedia, Loxosceles laeta* (Brazil), *L. laeta* (Peru), and *L. similis* (serially diluted 1/25 to 1/200). **(B)** ELISA showing the reaction of rMEPLox against anti-LALP-1, anti-LiHYAL, and anti-LiD (diluted 1/200) ELISA and values given are the means of duplicates. The absorbance of the samples was determined at 492 nm.

Antigenicity of rMEPLox is shown in Figure 5, by its cross-reactivity with rabbit antibodies elicited against *L. intermedia, L. laeta, L. gaucho*, and *L. similis* venoms or against individual recombinant toxins (LALP-1r, LiHYALr, LiD1r) from *L. intermedia* venom, tested by ELISA. The interaction results of rabbit anti-Loxoscelic venoms antibodies with rMEPLox are shown in Figure 5A. All specific loxoscelic antivenoms used in this study contains antibodies able to bind to rMEPLox coated in ELISA plates, although with different intensities. Examining the reactivity of anti-rLiD1, anti-LALP1r, anti-LiHYALr rabbit antibodies against rMEPLox (Figure 5B), it could be observed that all specific anti-toxins antibodies recognize rMEPLox, with higher intensity than the venoms.

**Figure 6:**
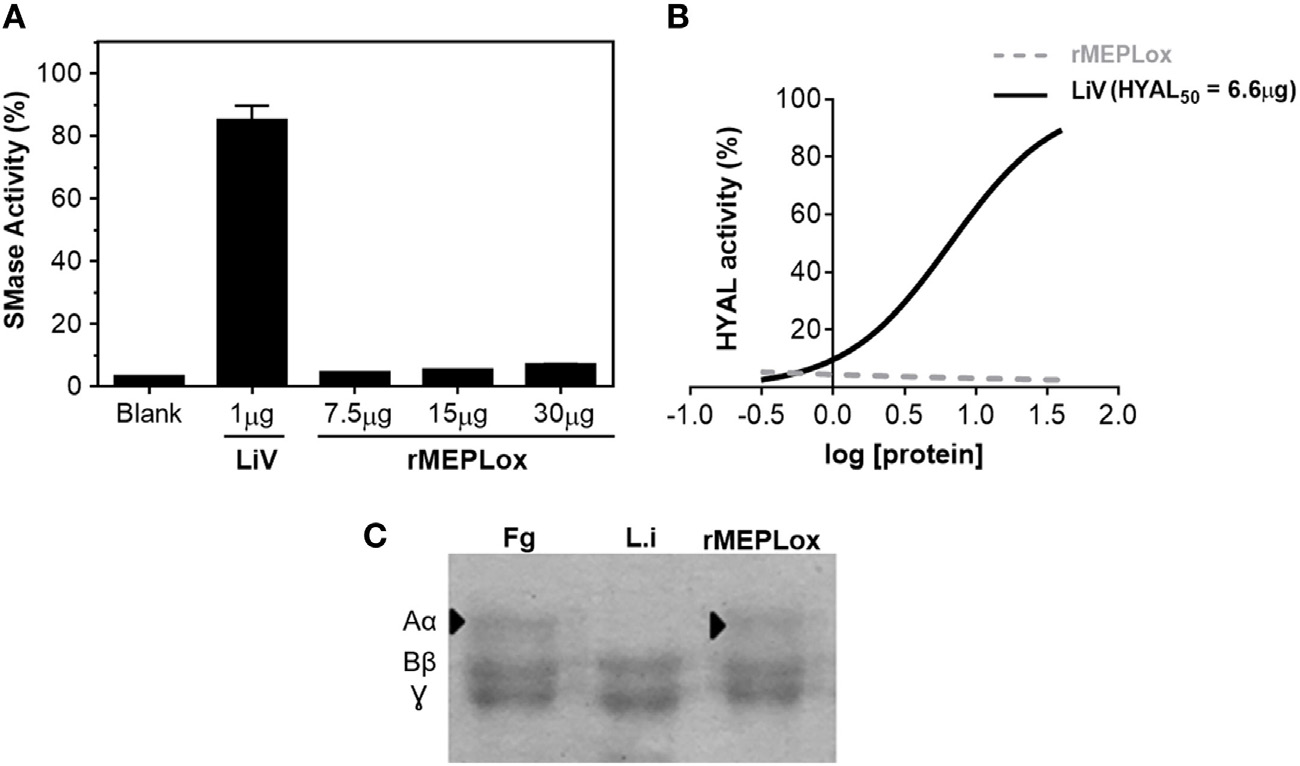
Enzymatic activities of rMEPLox. **(A)** Sphingomyelinase activity assay of rMEPLox at different concentrations (7.5, 15, and 30 μg), using AmplexRed^®^ Invitrogen Kit. One microgram of *Loxosceles intermedia* venom (LiV) was used as positive control. **(B)** Turbidimetric assay for detecting hyaluronidase (HYAL) activity. Serial dilutions of rMEPLox and LiV were used to determine the concentration capable of degrading 50% of the hyaluronic acid in the assay solution (HYAL_50_). **(C)** Fibrinogenolytic assay to assess proteolytic activity of rMEPLox. In the first lane, fibrinogen (Fg) showing its three characteristic bands, corresponding to α, β, and γ subunits. The second lane shows Fg previously incubated with LiV, which was capable of completely degrading subunit α. The third lane shows Fg previously incubated with rMEPLox, showing intact Fg bands.

One advantage of using a recombinant multiepitopic proteins instead of venom or toxins (either purified or recombinant) is the fact that epitopes, that correspond only to protein fragments, are more likely to be devoid of toxicity. To check if rMEPLox displays the enzymatic activities of *L. intermedia* venom toxins that originated its sequence, *in vitro* assays were performed. Figure 6 shows that rMEPLox do not have relevant sphingomyelinase (Figure 6A), HYAL (Figure 6B), or metalloproteinase (fibrinogenolitic) (Figure 6C) activities. For these enzymatic assays, *L. intermedia* venom (LiV) was used as positive control.

### Immunogenicity of rMEPLox

**Figure 7:**
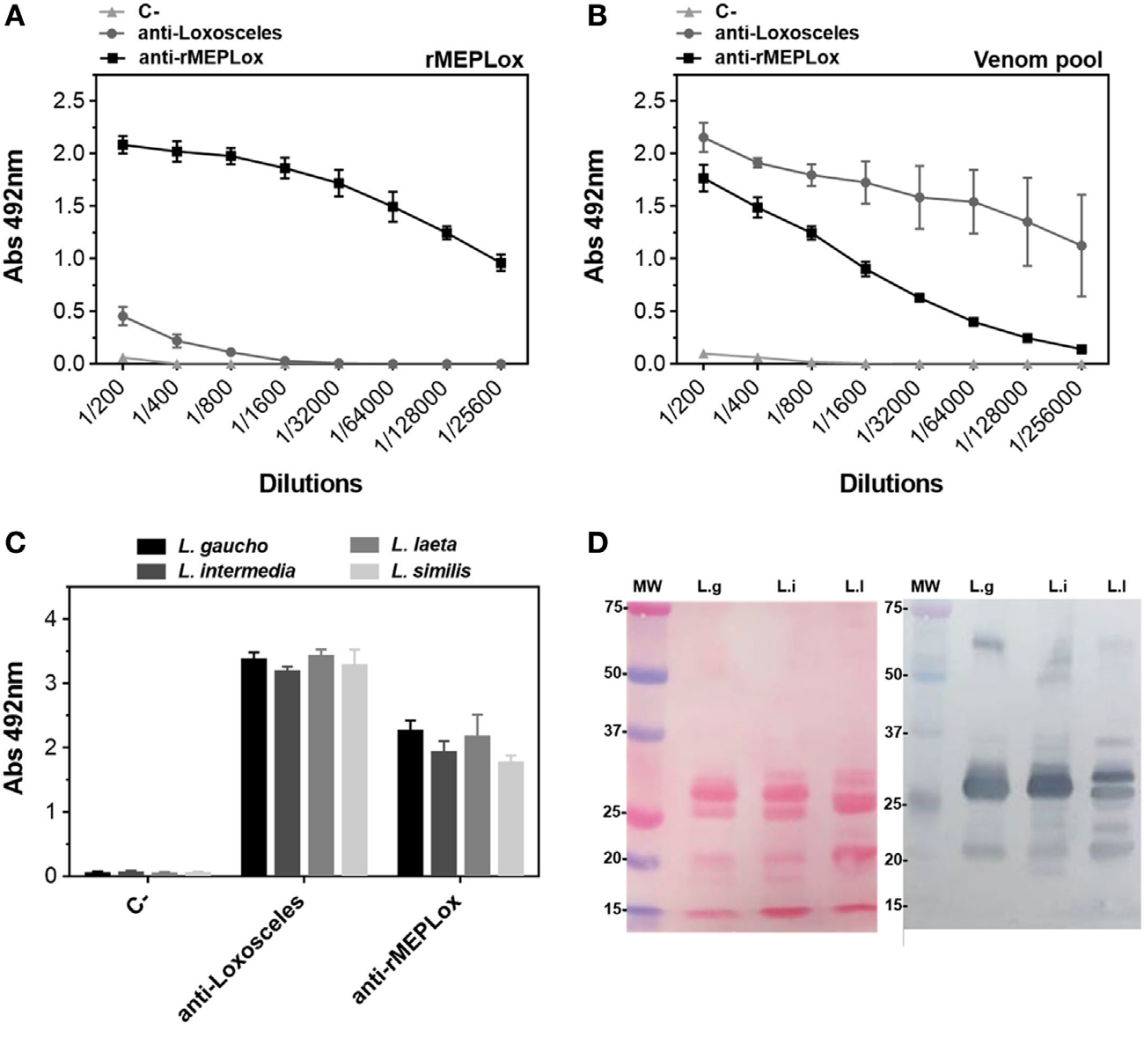
Immunoreactivity of anti-rMEPLox sera by ELISA. Plates were coated with either rMEPLox, a pool or individual *Loxosceles* venoms (5 μg/mL) and sera from rabbits immunized either with rMEPLox, a pool of *Loxosceles* venoms or only adjuvant were assayed by ELISA and values given are the means of duplicates. Samples absorbance was determined at 490 nm. **(A)** Reaction of rMEPLox with the tested sera, in serial dilution. **(B)** Reaction of coated pool of *Loxosceles* venoms with the tested sera, in serial dilution. **(C)** Reaction of coated individual loxoscelic venoms with the tested sera, in 1/400 dilution. Sera were tested by ELISA and values given are the means of trplicates. **(D)** At the left panel (Ponceau), the figure shows the profile of the transferred proteins. At the right panel, western blotting of loxoscelic venoms [*L. gaucho* (L.g); *L. intermedia* (L.i); and *Loxosceles laeta* (Ll)] probed with anti-rMEPLox rabbit sera 1/1,000 dilution.

To assess the capacity of rMEPLox to stimulate a protective immune response in model animals, rMEPLox vehiculated in Montanide adjuvant was used as immunogen in two rabbits. As a positive control, a pool of *L. intermedia, L. gaucho*, and *L. laeta* venom was used to immunize two more rabbits. The negative control rabbits received only Montanide, the adjuvant used in the other two groups. One week after the last injection, the reactivity of anti-rMEPLox, anti-*Loxosceles* venom pool (C+) and negative control serum samples toward rMEPLox, the pool of *Loxosceles* venoms used for immunization and individual venoms of *L. intermedia, L. laeta, L. gaucho*, and *L. similis* venoms were tested by indirect ELISA and immunoblot. Figure 7A shows the reactivity of rabbits’ sera against rMEPLox coated to ELISA plates. As expected, rMEPLox showed high reactivity with anti-rMEPLox antibodies, confirming its immunogenicity in eliciting specific antibodies. Rabbits that received the pool of *Loxosceles* venoms had lower antibody reactivity toward rMEPLox. As immunogen, rMEPLox generated antibodies that cross-reacted with the pool of *Loxosceles* venoms (Figure 7B). The cross-reactivity against individual *Loxosceles* species (*L. intermedia, L. laeta, L. gaucho*, and *L. similis* crude venoms), was also evaluated. Results of ELISA (Figure 7C) showed high antibody response against individual venoms for both immunized groups.

Analysis by western blotting (Figure 7D) showed that antibodies from rabbits immunized with rMEPLox recognized most components in the three tested *Loxosceles* venoms (*L. similis* reactivity was not assayed). While, as expected, components with molecular masses of ~60, 32–35, and 20–25 kDa which corresponded, respectively, to HYAL, SMase D, and metalloproteinases proteins in these venoms were recognized by anti-rMEPLox antibodies. These results agree with those obtained by ELISA.

### Neutralization Assays

Since antibodies capable of binding to SMase D, HYAL, and metalloproteinase loxoscelic toxins were detected in the produced anti-rMEPLox serum, the potential of these antibodies to neutralize *in vivo* and *in vitro Loxosceles* venom activities was assessed. Anti-rMEPLox IgGs were purified from immunized rabbit sera using a Protein-A-Sepharose column, to avoid interference of other serum components in the assays.

For *in vivo* neutralization assays, the ability of circulanting anti-rMEPLox rabbit antibodies to block the toxic activity of *L. intermedia* venom was tested by measuring the reduction of dermonecrosis induced by the venom in the immunized rabbits. This approach evaluated the vaccinal potential of this multiepitopic recombinant protein. Dermonecrosis was measured in rabbits, after injection of 10 μg of *L. intermedia* venom. As can be seen in Table 1 and Figure S1 in Supplementary Material, the pre-existent response from immunized rabbits with the *Loxosceles* venom pool or rMEPLox were able to effectively neutralize the venom main toxic effect. These immunized rabbits demonstrated 100% protection against dermonecrosis. Rabbits that received only adjuvant (negative control) were not at all protected.

**Table 1:** Measurement of dermonecrosis area (squared centimeters) caused by 10 μg of *L. intermedia* venom in immunized rabbits.

Dermonecrosis lesion area (cm^2^)			
Time (h)	6	24	72
Non-immune	2.77	9.04	9.90
Anti-*Loxosceles*	0	0	0
Anti-rMEPLox	0	0	0

To perform *in vivo* lethality neutralization assays, the median lethal dose (LD_50_) of *L. intermedia* venom was established as 10 μg of *L. intermedia* venom, for male BALB/c. A venom amount corresponding to 2.5 LD_50_, pre-incubated with 100 μL of anti-rMEPLox or pre-immune (PI) sera were injected to mice and deaths were recorded (Table 2). The lethality neutralization test showed that sera raised against rMEPLox can prevent death in envenomed animals as after 72 h of envenoming, 60% protection was observed. In control groups, there was 20% protection to the animals that received venom pre-incubated with PI serum and there was no protection to the animals that received only venom diluted in PBS.

**Table 2:** Lethality neutralization of *L. intermedia* venom by anti-rMEPLox serum.

Survival (%)							
Time (h)	0	12	24	36	48	60	72
Phosphate buffer saline	100	80	80	0	0	0	0
Pre-immune	100	80	80	20	20	20	20
Anti-rMEPLox	100	100	100	60	60	60	60

**Figure 8:**
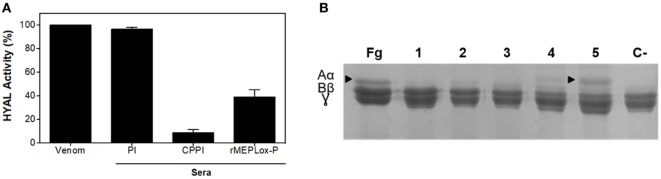
Neutralization of *Loxosceles intermedia* enzymatic activities by anti-rMEPLox antibodies. **(A)** Neutralization of hyaluronidase (HYAL) activity, measured in turbidimetric assay. *L. intermedia* venom (6.6 μg) was considered as 100% active. The same amount of venom was pre-incubated with either pre-immune sera (PI), commercial antivenom produced at CPPI or anti-rMEPLox sera (2 μL). **(B)** Neutralization of fibrinogenolytic activity by different amounts of rMEPLox antibodies. In the first lane, fibrinogen (Fg) showing its three subunits α, β, and γ intact. Lane 1 shows the degradation of the alpha chain when Fg is treated with 3 μg of *L. intermedia* venom. In the following lines, the venom was pre-incubated with concentrations of 2 (2), 10 (3), 40 (4), and 100 μg (5) of anti-rCpLi2 IgG. A negative control (C−) was made with venom pre-incubated with non-immune IgG.

For *in vitro* neutralization of hyaluronidase (HYAL) activity, the turbidimetric assay was performed. An amount of venom capable of degrading 50% of hyaluronic acid in the assay, previously established as 6.6 μg, was incubated in the complete absence of serum. This sample presented high enzymatic activity and was considered as 100% activity in this assay (Figure 8A). Pre-incubation of *L. intermedia* venom with pre-immune sera did not affect HYAL activity. Pre-incubation of *L. intermedia* venom with commercial anti-Loxocelic antivenom (CPPI) or with rabbit IgGs anti-rMEPLox (100 μg of each antibody) neutralized the HYAL activity of this spider venom. Commercial horse antivenom completely abolished HYAL activity, while anti-rMEPLox showed approximately 60% of inhibition.

To evaluate the neutralization of metalloproteinase activity, the assay of fibrinogenolysis was performed. Pretreatment of *L. intermedia* venom with anti-rMEPLox antibodies prevented fibrinogenolysis induced by the venom (Figure 8B). Absolute prevention of alfa-chain digestion can be observed with 100 μg of anti-rMEPLox (Figure 8B lane 5).

## Discussion

This study was motivated by our former observation that serum raised against a previously produced recombinant protein (rCpLi) containing three epitopes from LiD1, a dermonecrotic toxin from *L. intermedia* spider venom, displays reactivity with parent *Loxosceles* dermonecrotic toxins and shows *in vivo* and *in vitro* neutralizing capacity (22). This protein could also be efficiently used for antivenom production in horses (36).

In the present work, to confirm and enrich the possibilities raised by the anti-recombinant multiepitopic protein antibodies approach, nine B-cell epitopes of four major *Loxosceles* toxins were simultaneously expressed, generating a new, more complex recombinant multiepitopic protein which was used to immunize rabbits. Conformational epitopes could represent significant antigens for generation of neutralizing antibodies (37, 38). However, our previously work showed that the combination of a conformational epitope identified by phage display and two other linear epitopes could be employed to generate a protective immune response against *Loxosceles* venom (22, 36). Nevertheless, linear peptides should be evaluated as they do not always generate a protective immune response compared to native protein epitopes, which include conformational ones (39).

The new multiepitopic protein (rMEPLox) produced consists of conformational and linear B-cell epitopes: one conformational (NCNKNDHLFACW) and two linear (LGANSIETDVSFD DNANPEYTYHGIP and SKKYENFNDFLKGLR) of SMase D of *L. intermedia* (22), one linear epitope (DFSGPYLPSLPTLDA) of SMase-I from *L. laeta* (28), three linear epitopes (SLGRGCTDFGTILHE, ENNTRTIGPFDYDSIMLYGAY and KLYKCPPVNPYPGGIRPYVNV) of LALP-1, and then two linear epitopes (NGGIPQLGDLKAHLEKSAVDI and ILDKSATGLRIIDWEAWR) of LiHYAL. The epitopes from metalloproteinase LALP-1 and HYAL LiHYAL from *L. intermedia* spider venom were mapped in the first part of this work, by the SPOT method. This methodological approach was found extremely useful to rapidly map continuous epitopes (40–42). We limited the number of epitopes used in the rMEPLox to avoid unknown folding which could hide epitopes of interest in the recombinant protein structure. In our view, a smaller protein is a better strategy if the aim is to ensure that all the epitopes are exposed for recognition and generation of antibodies.

We demonstrated that the generated protein expressing B-cell epitopes of *L. intermedia* and *L. laeta* toxins is a non-toxic molecule. When compared with *L. intermedia* crude venom or its purified toxins, rMEPLox did not show sphingomyelinase, hyaluronidese, or metalloproteinase enzymatic activities. SMase D family (also known as the dermonecrotic toxins), metalloproteases, and HYALs are known classes of brown spider toxins. SMases D and metalloproteases can induce inflammatory response, dermonecrosis, hemolysis, thrombocytopenia, and renal failure (43). The functional role of HYAL toxin as a spreading factor in loxoscelism has also been demonstrated (12). However, the biological characterization of other toxins such as insecticidal peptides, serine proteases, allergen-like, and protease inhibitors in the context of *Loxosceles* venom remains to be further elucidated. Once other classes of *Loxosceles* toxins have proven to be relevant to envenoming, epitopes derived from their sequences can be incorporated to the multiepitopic recombinant protein, simply by inserting their respective nucleotidic sequence to the vector that already contains rMEPLox. This methodology was successfully used herein to incorporate new epitopes to a previously produced multiepitopic protein rCpLi and shows the potential of continuous improvement of this recombinant protein as antigen.

ELISA assays revealed the antigenicity of rMEPLox by its reactivity with rabbit antibodies against *L. intermedia, L. laeta, L. gaucho*, and *L. similis* venoms or against recombinant individual *L. intermedia* toxins (anti-rLiD1, anti-rLALP-1, and anti-rLiHYAL). The ELISA findings imply, as expected, that rMEPLox shares similar antigenic identities or common epitopes across Brazilian *Loxosceles* spider venoms’ toxins studied. It possibly contains immunogenic constituents which are not similar in composition to those found in the venom of *L. laeta* from Peru, as indicated by its lower reactivity. In fact, corroborating our results, Guimarães et al. (44) shows intraspecific differences in the protein profile of Brazilian and Peruvian *L. laeta* venoms. The immunological cross-reactivity of anti-rMEPLox antibodies with other *Loxosceles* spider antigens, like observed by ELISA and Western blot, is expected since toxins of Brazilian *Loxosceles* spiders form a family of structurally related proteins. Indeed, these antibodies achieved similar titers in ELISA assays against *L. intermedia* and *L. gaucho, L. laeta*, and *L. similis*, indicating that it is likely that efficient neutralization can be achieved as well. This indicates that rMEPLox may be an efficient antigen to produce antivenom against Brazilian *Loxosceles* venom. However, in order to produce a common American antivenom, for a border application, further studies must be developed to incorporate additional relevant epitopes from other *Loxosceles* species to the antigen.

The main objective of this work was to use a multiepitopic recombinant protein (rMEPLox) to obtain rabbit antibodies with predetermined specificity, able to neutralize the dermonecrotic and lethal effects of *L. intermedia* spider venom, which is the spider of major medical importance in Brazil. This goal has been reached, since we have shown that anti-rMEPLox antibodies raised against epitopes of four *Loxosceles* toxins can neutralize sphingomyelinase, proteolytic, and HYAL *in vitro* enzymatic activities. The epitopes that compose rMEPLox have been identified using different approaches. The three that are from SMase D from *L. intermedia* and the one from SMase D of *L. laeta* have their neutralizing potential previously evaluated by *in vivo* and *in vitro* assays (22, 28, 36). The other epitopes were mapped in this work and, although their neutralizing capability were not determined before, they were identified using the same methodology employed to map other relevant epitopes using sera from immunized animals (18, 19).

Furthermore, *in vivo*, rMEPLox can protect immunized rabbits from *L. intermedia* deleterious effects and BALB/c mice against the lethal effect of the of *L. intermedia* venom. However, since the rabbits were not hyperimmunized as horses generally are for producing antivenom sera, mice *in vivo* neutralization was not complete. As previously seen by Figueiredo et al. (36), more immunizations doses may be necessary to produce a serum with a highly neutralization effect, even when whole venom is used. This was also observed by Zenouaki et al. (45), who reported an enhancement in antibody titer after a boost immunization with a synthetic analog of a toxin from the scorpion *Androctonus australi*s hector. Remarkably, protection of *L. intermedia* venom toxic effects given by pre-existent circulating IgGs against rMEPLox was comparable to that obtained by the mixture of *L. laeta,L. intermedia*, and *L. gaucho* venoms antigens. Anti-rMEPLox as well as anti-loxoscelic venoms antibodies provided full protection (100%) against dermonecrosis, which is the main complication of *Loxosceles* envenoming. This result shows that one single non-toxic protein can evoke an immune response with specificities similar to the one elicited by complex mixtures of toxic proteins, with the potential of substituting crude *Loxosceles* venoms in antivenom production. However, it was not tested yet if anti-rMEPLox antibodies are equally effective in neutralizing other *Loxosceles* species *in vivo* toxic effects.

These results show that by rapidly mapping epitopes and using them to compose a recombinant multi-epitope immunogen to obtain specific anti-epitopes antibodies is an efficient strategy to achieve venom neutralization. This approach can be considered for straightforward preparation of *Loxosceles* antivenoms, diminishing or even dismissing the use of crude venom. This recombinant multiepitopic protein is a non-toxic molecule and, therefore, represents a safer immunogen concerning animal welfare in antivenom production. In addition, it has flexibility to be produced in large scale and can be modified using simple approaches to incorporate new insights in venom toxicity and epitope refining.

## Ethics Statement

Experimental protocols were performed after approval by the Ethics Committee in animal Experimentation of Federal University of Minas Gerais (388/2017-CETEA/UFMG).

## Author Contributions

SL, CG-D, DO, VF, DT-S, and RA performed the experiments. TM, SV, JM, EK, and CC-O conceived and designed the experiments. SL, CG-D, FC-O, and LF analyzed the data and prepared the figures. CG-D and CC-O were responsible for guiding, supporting the experiments and drafting the manuscript. All the authors revised the work and gave final approval of the version to be published.

## Conflict of Interest Statement

The authors declare that there are no conflicts of interest.
