# Supplementary material for: Recombinant Protein Containing B-Cell Epitopes of Different Loxosceles Spider Toxins Generates Neutralizing Antibodies in Immunized Rabbits
Source: Front Immunol. 2018 Apr 3;9:653. doi: 10.3389/fimmu.2018.00653 (PMC5891610; doi:10.3389/fimmu.2018.00653)
Supplement: Supplementary file 1 [file Image_1.PDF]

Supplementary Material:

Recombinant protein containing B-cell epitopes of different *Loxosceles* spider toxins generates neutralizing antibodies in immunized rabbits

S. de Almeida-Lima, C. Guerra-Duarte, F. Costal-Oliveira, T. Melo Mendes, L.F.M. Figueiredo, D. Oliveira, R. A. Machado de Avila, V. Pereira Ferrer, D. Trevisan-Silva, S.S. Veiga, J.C. Minozzo, E. Kalapothakis, C. Chávez-Olórtegui*

*Correspondence: Dr. Carlos Chávez-Olórtegui
Departamento de Bioquímica e Imunologia, Instituto de Ciências Biológicas, Universidade Federal de Minas Gerais (UFMG), Avenida Antônio Carlos, 6627, Belo Horizonte, Brazil. CEP: 31270-901.
Telephone number: 55 31 3409 2625
Fax number: 55 31 3409 2613
E-mail: olortegi@icb.ufmg.br

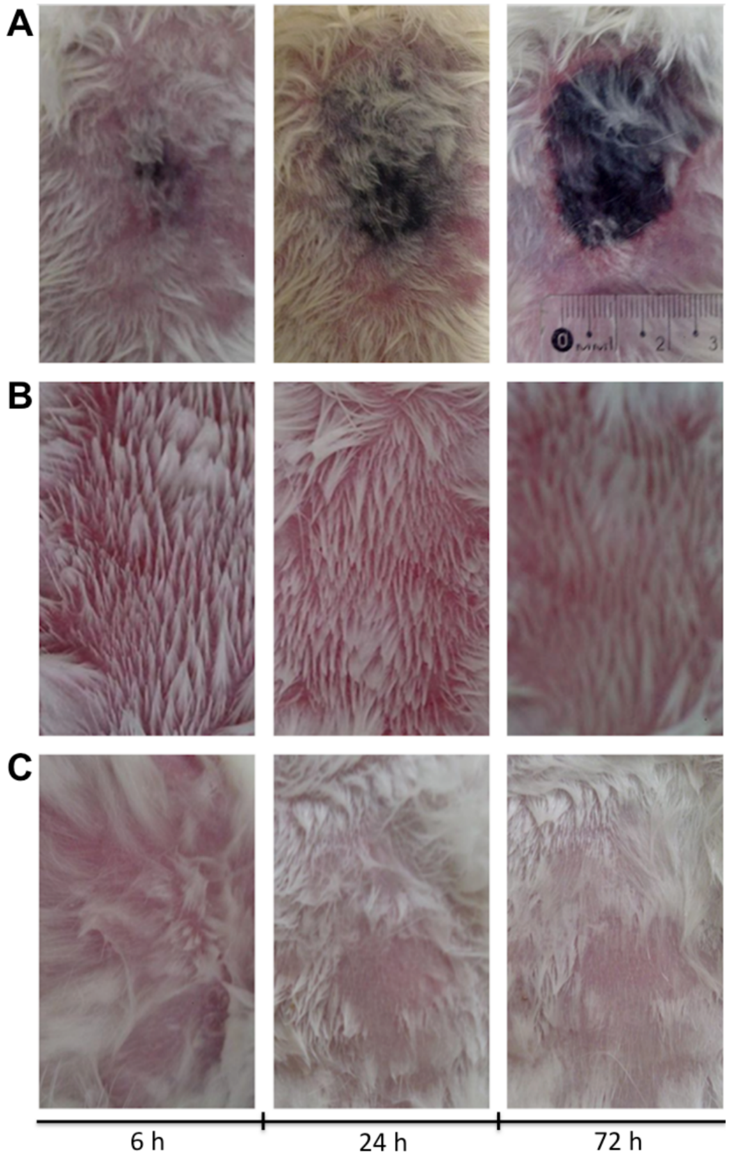

Figure S1. ***In vivo dermonecrosis neutralization assay*****.** Dermonecrotic lesions were induced in immunized and control rabbits by *Loxosceles intermedia* venom (10 µg). (A) Non-immune rabbit. (B) Rabbit immunized with pool of loxoscelic venoms. (C) Rabbit immunized with rMEPLox-Tox. Macroscopic visualization of dermonecrosis was evaluated 6, 24 and 72h after the challenge.
